# From Distress To Disruption in Early Childhood: Time-Varying Associations Between Internalizing and Externalizing Problems, Child Sex and Prenatal Cocaine Exposure

**DOI:** 10.1007/s10802-025-01369-z

**Published:** 2025-10-21

**Authors:** Miglena Y. Ivanova, Rina D. Eiden, Danielle M. Seay, Kristin J. Perry, Ashley N. Linden-Carmichael

**Affiliations:** 1 Department of Psychology, The Pennsylvania State University, University Park, PA USA; 2 Edna Bennett Pierce Prevention Research Center, The Pennsylvania State University, University Park, PA USA; 3 The Social Science Research Institute, The Pennsylvania State University, University Park, PA USA; 4 Department of Counseling Psychology and Human Services, University of Oregon, Eugene, OR USA

**Keywords:** Internalizing, Externalizing, Time-Varying Effect Modeling, Cocaine Exposure

## Abstract

Evidence of internalizing (INT)-to-externalizing (EXT) directional effects (e.g., acting out/masked depression hypotheses) has been commonly found in older youth. However, this relationship may still be present but fluctuate more dynamically during early development, a period of significant cognitive and self-regulatory changes. Moreover, dynamic codevelopment has not been well studied in samples at elevated risk, including those due to prenatal cocaine exposure. There is some evidence that prenatal cocaine exposure increases risk for EXT, but findings have been mixed. One possible explanation may be that INT may codevelop with EXT in higher-risk contexts, and most studies do not consider codevelopment of these problems. We utilized time-varying effect modeling to investigate the age-varying effect of INT on concurrent EXT across early childhood (1.5–6.5 years) and whether associations varied as a function of sex and prenatal cocaine exposure. The sample was primarily low-income and Black (*N* = 198; 49% males; 74% Black; 71% receiving federal assistance), oversampled for prenatal cocaine exposure using a case-control design. Results identified the strongest regression effect of INT on EXT during the toddlerhood/preschool period (2.5–4.5 years), especially for male children. No differences were observed by cocaine exposure. Findings suggest that INT problems are most strongly associated with increased EXT difficulties at earlier ages, which may be a sensitive period during which targeting INT may potentially prevent long-term co-occurring problems.

**Supplementary Information:**

The online version contains supplementary material available at 10.1007/s10802-025-01369-z.

## Time-Varying Fluctuations in the Associations Between Internalizing and Externalizing Problems: Codevelopment and Sex Differences During Early Childhood

Ample evidence links early co-occurring problems to subsequent maladaptive outcomes (e.g., poorer social and school outcomes, risky behaviors, and psychopathology; Campbell, et al., 2010). Thus, it is critical to understand how broad internalizing (INT; e.g., depression/anxiety) and externalizing (EXT; e.g., aggression/hyperactivity) domains may codevelop, especially for children already at higher risk for suboptimal developmental outcomes. During early childhood, children commonly follow a curvilinear pattern of EXT problems (increase in problems during early to late toddlerhood, followed by a decrease in preschool years; Alink et al., 2006), whereas INT problems remain stable at low-to-moderate levels (Lavigne et al., 2001). High rates of temporal co-occurrence (i.e., occurring at the same time) have been reported with different codevelopmental processes’ explanations (e.g., *acting out*,* transactional models*; Gjone & Stevenson, 1997; Glaser, 1967). However, the nuanced dynamics between the two domains have been less commonly explored, especially among children at higher risk based on biological and environmental vulnerabilities.

Rapid changes in fundamental skills (e.g., self-regulation and independence) and relationships mark the formative early childhood years (Colson & Dworkin, 1997; Davies et al., 2013). Thus, it is possible that the magnitude of the INT-EXT association fluctuates more rapidly (i.e., *time-varying association*). Ages at which this association is strongest may represent a sensitive period during which prevention/intervention efforts may be particularly effective in targeting the long-term development of co-occurring problems. Furthermore, gender socialization theories (Endendijk et al., 2018; Ostrov & Godleski, 2010) suggest that the magnitude of the INT-EXT association may vary between male and female children who are socialized differently on how to express emotions in socially acceptable ways (Cole, 1985; Zahn-Waxler et al., 2000). Finally, fetal stress posed by prenatal substance exposure, such as cocaine, has been associated with elevated behavior problems, particularly in the EXT domain (Minnes et al., 2010; Molnar et al., 2014), although results have been mixed (Accornero et al., 2002). The goals of the current study were to examine (1) the time-varying effect of INT on EXT problems during early childhood in a sample at elevated risk, (2) how it may differ between male and female children, and (3) whether it varies as a function of prenatal cocaine exposure (PCE).

### Overview of Early Childhood Codevelopment

There are different theoretical frameworks for understanding co-occurrence among INT and EXT problems. The *general predisposition* framework proposes that a shared etiology may explain the co-occurrence of INT-EXT problems (e.g., general psychopathology ‘p’ factor; Gjone & Stevenson, 1997; Lahey et al., 2012). Second, *directional theoretical explanations* focus on the cross-domain dynamics and directional processes of how problems in one domain may be associated with subsequent increases in the other domain. One such common explanation when considering the directional effects of INT on EXT problems is the *acting-out* (also *masked depression*) explanation. This hypothesis suggests that children with INT problems may show increases in EXT problems through “acting out” their internal distress, “masking” their depression (Carlson & Cantwell, 1980; Glaser, 1967). Few studies have investigated these associations through frequent assessments during early childhood, especially among children at high risk due to the combination of biological/environmental vulnerability posed by PCE and structural risks, including those posed by lack of resources related to low income.

In predominantly lower-risk samples, evidence supporting the *acting-out* explanation comes from studies conducted at later ages and with larger time lags between assessments (Beyers & Loeber, 2003; Chen & Simons-Morton, 2009). However, INT problems may present as immediate increases in EXT via ‘acting out’ internal distress (Luby et al., 2003), rather than only affect EXT in future assessments (i.e., after months/years). This may explain the failure to find support for the acting-out hypothesis during early childhood, when the effects of internal distress may be more immediate rather than cumulative. The strength of the time-varying associations may differ by age. We would expect a decline in the association between INT and EXT during early childhood as self-regulation improves.

### Overview of Sex Differences in Codevelopment

There have been conflicting findings regarding sex differences in early childhood. Some studies report higher INT among girls, although evidence is mixed (Gutman & Codiroli McMaster, 2020; Zahn-Waxler et al., 2008). However, there is more robust evidence that boys display greater EXT (Archer, 2004; Zahn-Waxler et al., 2015). Distinct biological processes and associated gene expression between male and female children may partially contribute to sex differences in INT and EXT behaviors (for a review, see Zahn-Waxler et al., 2015). However, little is known about sex differences in codevelopmental processes. Moreover, it is challenging to differentiate biological from acculturation effects, given that both contribute to gender-typed behaviors (i.e., behaviors that differ by gender; Endendijk et al., 2018), especially during early childhood. Gender socialization theories (Endendijk et al., 2018; Ostrov & Godleski, 2010) suggest that the magnitude of the INT-EXT association may vary between male and female children, who are socialized differently on how to express emotions in socially acceptable ways (Cole, 1985; Zahn-Waxler et al., 2000). As a result of biological and/or socialization influences, *girls may be more likely to express distress inward* as INT problems, whereas *boys may express distress outward as EXT problems* (Lee et al., 2022; for meta-analysis and reviews, see Archer, 2004; Chaplin & Aldao, 2013). Further, the ‘gender paradox’ theory suggests a higher co-occurrence of INT and EXT problems when children display gender-incongruent behaviors (Loeber & Keenan, 1994; Wiesner & Kim, 2006). For example, boys who display more INT problems, which are more gender-incongruent, may begin to display more EXT problems over time. Thus, we need to consider early sex differences when gender socialization influences are pronounced (Bussey & Bandura, 1999; Martin & Ruble, 2010).

### Overview of PCE and Behavior Problems

The field of behavioral teratology and the developmental origin of health and disease model (e.g., Werboff & Gottlieb, 1963) have long highlighted the importance of prenatal environment in shaping and predicting developmental outcomes across domains. PCE is associated with disruptions in the dopaminergic and monoaminergic systems, resulting in alterations in arousal, attention, and stress reactivity regulatory systems (e.g., Lester & Padbury, 2009). These difficulties with regulation may disrupt the expected decrease in the INT-EXT association across early childhood. Given that, during toddlerhood, children are still learning how to self-regulate and verbalize internal distress (Davies et al., 2013), it is also possible that children are more likely to express their internal distress as EXT problems. This is in line with some explanations of codevelopment (Carlson & Cantwell, 1980; Glaser, 1967). However, to our knowledge, researchers have not examined codeveloping INT-EXT associations in early childhood among children with substance exposure or examined how PCE may moderate the developmental progression of this association. Thus, we examined changes in the stability, direction, and magnitude of the time-varying effect of INT on EXT problems over time and examined if this association varies for demographically similar children with PCE compared to no PCE.

### Overview of Methodological Considerations

Most prior work has examined the codevelopment of INT and EXT problems using structural equation and latent class/mixture modeling frameworks (Burt & Roisman, 2010; Fanti & Henrich, 2010; Gilliom & Shaw, 2004). While useful for examining longitudinal codevelopment of INT and EXT, such modeling approaches traditionally fix coefficient estimates, thereby restricting examinations of precise ages during which associations are strongest. One novel approach that can enable nuanced examinations of fluctuations in associations is time-varying effect modeling (TVEM; Lanza & Linden-Carmichael, 2021). TVEM is an extension of linear regression that permits estimations of how the dynamic relationship between two variables (e.g., INT and EXT) may change across developmental age and as a function of individual-level factors (e.g., PCE, sex). By allowing coefficients to vary non-parametrically as a flexible function of developmental age, we can identify sensitive periods of strong INT-EXT associations during early childhood’s rapid development.

### Present Study

This study had three primary aims. First, we investigated time-varying fluctuations in the INT-EXT association by examining the concurrent regression effect of INT on EXT in a high-risk sample of children exposed to PCE from under-resourced, low-income environments. According to the acting-out hypothesis, young children often have limited ability to communicate and self-regulate internal distress, a difficulty that may be especially pronounced in children at higher risk. Therefore, we hypothesized that the effect of INT on EXT problems would be strongest in toddlerhood but would slowly decrease over time as children develop more self-regulation (H_1_). Second, we examined whether the time-varying association between INT and EXT problems differed by child’s sex. Due to the current state of the literature on gender socialization and gender-incongruent behaviors (e.g., INT problems in male children associated with more EXT), we hypothesized that INT would be more strongly associated with concurrent EXT problems in male than in female children (H_2_). Given the novelty of this aim, we did not hypothesize about the timing of significant sex differences. Finally, there is limited literature on the effects of PCE on INT-EXT associations, which we conceptualized as both a biological and environmental risk factor. As such, we did not have specific hypotheses about how these associations may vary as a function of PCE, but examined whether the association differed by PCE, while also accounting for other substance use, as PCE often co-occurs with other substance exposures.

## Method

### Participants

The full sample consisted of 216 mother-child dyads (51% female) who participated in an ongoing longitudinal study examining the effects of PCE on child development. We recruited mothers between 2001 and 2006 from two urban hospitals primarily serving lower-income families of color. Exclusionary criteria were maternal age less than 18 years, use of illicit substances other than cocaine or marijuana during pregnancy, multiple births, and significant medical problems or complications for the infant. Of the 216 children, 116 were in the PCE group with cocaine exposure in utero. PCE and non-PCE families were matched based on race/ethnicity, infant sex at birth, and maternal education, with 75.9% of the full sample reporting some level of other substance use and 52.3% reported using more than one substance. For the analytic sample, 198 families (50% female; *n* = 107 PCE group) who had data on behavior problems at least once during early childhood were included; thus, 18 families were excluded from analyses. Most biological mothers identified as Black (74.2%), ranging 18 to 42 years of age at delivery (*M* = 29.58, *SD* = 6.07). At the time of recruitment and first visit (4–8 weeks after delivery), most mothers had a high school or below education (70.0%), were receiving federal assistance (76.6%), and were never married (67.7%).

### Procedures

This study was approved by the institutional review board at the University at Buffalo. Two weeks after delivery mothers were contacted to schedule their first laboratory visit. Follow-up assessments were conducted with the primary caregiver, defined as the person with legal guardianship of the child at that time (by early school age, about 30% of children had been in foster/kinship care at least once). The first and subsequent laboratory visits consisted of a combination of interviews, observations of caregiver-child interactions, and in-person assessments. Lab assessments were conducted at 1, 7, 13, 24, 36, and 48 months of child age and early school age (*M* = 66.54, *SD =* 4.18, sample range = 57.82–78.03). There was more variability in the timing of early school age assessments, which were conducted approximately three months after kindergarten entry to avoid the effects of immediate adjustment to formal schooling. Children were assessed at age corrected for prematurity until the 24-month assessment and their chronological ages were used in analyses for time-varying associations (see below). In addition, questionnaire assessments/telephone interviews were conducted every six months between laboratory visits with measurement of child behavior problems beginning at 18 months of child’s age. Biological mothers were interviewed during the first laboratory visit to obtain information about prenatal substance use for all participants regardless of custody. Informed consent was collected from caregivers at all time points. Participants were compensated for their participation in the form of checks, gift certificates, and child toys at each time point, with the amount increasing over time.

### Measures

#### Child’s Age

Child’s age was measured as the child’s chronological age in months at the time of each assessment, calculated from days since birth, and converted into months.

#### Behavior Problems

Caregiver reports of child behavioral problems were obtained during the 18-, 24-, 30-, 36-, 42-, 48-, 54-month, and early school-age assessments using the 1½−5 version of the Child Behavior Checklist (CBCL; Achenbach & Rescorla, 2000). The CBCL comprises 100-items on a 3-point response scale ranging from “not true” (0) to “very true” (2). Behavior ratings yield two domains of INT and EXT problems. Across the eight assessment points, Cronbach’s alphas for the INT problem scale (36 items) ranged from 0.86 to 0.92 and from 0.91 to 0.94 for the EXT scale (24 items). Total raw scores for INT (sample range = 0–45) and EXT problems (sample range = 0–46) were used in analyses (Table 1). Higher raw scores indicate more problems. CBCL has been found a highly reliable and valid measure, including in early childhood (for review see, Halle & Darling-Churchill, 2016) and has demonstrated longitudinal measurement invariance in early childhood for partial measures of the CBCL scales (Colder et al., 2002).

**Table 1:** Means and standard deviations of internalizing and externalizing problems

Subscale	Total		Girls		Boys		Cocaine-exposed		Non-exposed	
	*M*(*SD*)	*Borderline/* *Clinical %*	*M*(*SD*)	*Range*	*M*(*SD)*	*Range*	*M*(*SD*)	*Range*	*M*(*SD*)	*Range*
**18 Months**										
Internalizing	8.09(7.00)	7.2/10.8	7.79(6.72)	0–35	8.38(7.28)	0–30	8.71(7.23)	0–35	7.38(6.70)	0–29
Externalizing	12.42(8.42)	7.8/9.6	11.41(7.46)	0–33	13.38(9.18)	0–37	12.42(8.20)	0–33	12.42(8.72)	0–37
**24 Months**										
Internalizing	7.66(6.64)	8.2/7.6	8.15(6.49)	0–34	7.18(6.80)	0–36	8.28(6.71)	0–34	6.95(6.53)	0–36
Externalizing	12.39(8.81)	7.6/8.2	12.43(8.73)	0–41	12.34(8.95)	0–40	12.81(8.53)	0–41	11.90(9.16)	0–40
**30 Months**										
Internalizing	7.60(6.62)	10.7/5.9	8.25(7.01)	0–38	6.98(6.20)	0–25	7.66(7.42)	0–38	7.54(5.55)	0–24
Externalizing	13.52(9.27)	10.7/13.0	12.74(8.24)	0–35	14.27(10.16)	0–40	12.99(8.38)	0–37	14.17(10.27)	0–40
**36 Months**										
Internalizing	7.70(6.50)	10.9/7.3	8.01(5.99)	0–27	7.40(6.97)	0–36	7.74(6.16)	0–27	7.66(6.91)	0–36
Externalizing	12.53(8.89)	8.5/8.5	12.05(7.70)	0–38	12.99(9.92)	0–44	12.52(8.40)	0–38	12.53(9.47)	0–44
**42 Months**										
Internalizing	7.95(7.36)	5.5/10.9	8.34(7.50)	0–37	7.58(7.25)	0–30	8.32(7.92)	0–37	7.50(6.62)	0–37
Externalizing	13.53(10.25)	8.5/14.5	12.86(8.97)	0–36	14.18(11.37)	0–45	13.42(9.68)	0–37	13.68(10.99)	0–45
**48 Months**										
Internalizing	7.44(7.43)	5.6/9.9	8.01(7.22)	0–37	6.86(7.63)	0–41	7.89(7.51)	0–37	6.92(7.35)	0–41
Externalizing	10.94(8.82)	6.8/8.0	11.23(8.59)	0–40	10.64(9.09)	0–45	11.20(8.90)	0–40	10.64(8.78)	0–45
**54 Months**										
Internalizing	7.41(7.33)	3.7/8.0	7.66(7.31)	0–45	7.15(7.39)	0–39	**8.67(8.27)**	**0–45**	**6.03(5.90)**	**0–45**
Externalizing	10.96(9.14)	3.7/8.6	10.71(8.71)	0–46	11.22(9.61)	0–44	11.79(9.44)	0–46	10.06(8.79)	0–44
**60 Months**										
Internalizing	7.44(8.20)	9.0/8.4	8.24(8.83)	0–42	6.69(7.55)	0–43	7.99(8.98)	0–42	6.79(7.20)	0–43
Externalizing	9.90(8.40)	4.2/6.0	9.83(8.53)	0–34	9.97(8.33)	0–41	10.07(8.41)	0–34	9.70(8.44)	0–41

Significant differences are bolded. Borderline or clinical INT-EXT levels for youth in our sample are presented in percentages.

#### Prenatal Cocaine Exposure (PCE)

In addition to the health screener, medical record review, and hospital urine screens, mothers were administered the Timeline Follow-Back Interview (TLFB; Sobell et al., 1986) for all potential substances, including cocaine. The TLFB is a valid and reliable method of obtaining daily data on substance use with good test–retest reliability (Brown et al., 1998). Participants were provided with a calendar and asked to identify personal events (i.e., holidays, birthdays, vacations) throughout their pregnancy as anchors to aid recall. Information derived from the TLFB was used to calculate cocaine use frequency (i.e., number of days cocaine was used per week).

To supplement the self-report measure, PCE was also measured using biological samples. Mother-infant urine toxicology conducted at delivery was extracted from medical records and maternal hair samples were collected at the first laboratory visit. Approximately 90% of dyads had urine and hair samples available for analysis. Urine samples were screened for the level of metabolites of cocaine and other substances in maternal or infant urine. If the quantity of metabolites was > 300 g/ml, urine was rated as positive for drug use. Hair samples were assayed by Psychemedics Corporation using Radioimmunoanalyses (RIAH; Magura & Kang, 1996). Hair samples were screened for cocaine, followed by a gas chromatography/mass spectrometry confirmation for positive cocaine screens. While urine toxicology may detect cocaine use up to approximately 2 to 3 days after use (Moeller et al., 2008), hair grows at an average rate of 0.5 inch each month; thus, an average of 5 inches of hair length reflects substance exposure across pregnancy.

Participants were assigned to the cocaine exposure group (= 1) if any of these indices were positive for cocaine: maternal self-report during screening at delivery or TLFB, medical record review at delivery, maternal hair analysis, and maternal and infant urine toxicology. Of the 198 mothers included in this study, 107 mothers were included in the PCE group, and 91 in the control group. Of the 107 mothers in the PCE group, 54 mothers both self-reported use and had a positive biological sample; 31 mothers did not report cocaine use but tested positive; 22 mothers endorsed use but did not have a positive biological sample (quantity of use TLFB data only available for 12 of those). The average number of days per week cocaine was used was 0.51 (SD = 1.24; range 0–6.63). Although our binary grouping approach does not reflect the severity of cocaine use, it allowed us to account for discrepancies between self-reports and biological samples, maximizing the use of available PCE data, is the norm for many studies of PCE (for review, see Eiden et al., 2023).

#### Potential Covariates

Based on previous literature, as well as results from the current sample (Authors, Year), we tested models controlling for other prenatal substance exposure and cumulative environmental and sociodemographic risks during the first year of a child’s life (see Table 2). See Supplemental Materials for more information on covariates.

**Table 2:** List of measures for control variables tested in models

Covariate	Measures used	Computation	Reporter/Child age at assessment
**Other** **Prenatal Substance Exposure** **(PSE)**		**Proportion of average scores**	**Self-report during first lab visit** **(4–8 weeks)**
Prenatal substance use (cigarette, alcohol, cannabis)	Timeline Follow-Back Interview (TLFB; Sobell et al., 1986)	average of weekly amount of cannabis, cigarettes, and alcohol	
**Cumulative Environmental Risk (ENV)**		**Sum of individual dummy-coded ENV scores**	**Self-report during 3x child visits** **(1-**,** 7-**,** and 13-months)**
Caregiver psychological functioning	Brief Symptom Inventory (BSI; Derogatis, 1993)	1 = for score 1 SD below the M	
Caregiver exposure to violence	Timeline Follow-Back Interview (TLFB; Fals-Stewart et al., 2003)	1 = for any violence	
Caregiving instability	Structured Clinical Interview (SCI; Platzman et al., 2001)	1 = for ≥ 2 experiences of caregiver instability (e.g., separation from primary caregiver, changes in caregiving adults, and living environment)	
Postnatal substance use (cigarette, alcohol, cannabis, cocaine use)	Timeline Follow-Back Interview (TLFB; Sobell et al., 1986).	1 = for average score > 10 cigarettes/day 1 = for average score > 4 drinks/day 1 = for average score ≥ 1 joint/day 1 = for any cocaine	
**Sociodemographic Risk** **(SRD)**		**Sum of individual dummy-coded SDR scores**	**Self-report during first lab visit** **(4–8 weeks** **)**
Caregiver race*	Demographics	1 = if non-White race* (84.2%)	
Low caregiver education	Demographics	1 = if not received a high school diploma or equivalent (39.5%)	
Single parenthood	Demographics	1 = never married (66%)	
Low income-to-needs ratio	Demographics	1 = income-to-needs ratio in lower quartile (25.4%)	
Occupational status	Hollingshead scale (Hollingshead, 1975)	1 = occupation status in lower quartile (41.2%)	

* Caregiver non-White race or ethnicity was included in the cumulative SDR score as a proxy for structural barriers, disparities, racial discrimination, and bias faced by people of color.

### Analytic Plan

To examine our study aims, we used TVEM (Lanza & Linden-Carmichael, 2021). TVEM allows the intercept and regression coefficients to vary flexibly as non-parametric functions of time. By estimating the time-varying association between INT and EXT problems (*EXT*_*i*_ = *β*_*0*_(*t*) + *β*_*1*_(*t*)*INT*_*i*_ + ∈_*i*_*)*, we obtain values for the *intercept* (*β*_*0*_; mean level of one domain of problems across time, accounting for problems in the other) and *slope* (*β*_*1*_; regression coefficient function for the time-varying effects of one domain of problems on the other, controlling for any covariates). Second, we performed a time-varying moderation to test whether the time-varying INT-EXT association varied as a function of child’s sex over time (0 = female, 1 = male). Similar to standard practices in regression, if an interaction was significant at any timepoint (95% CI did not include zero), we conducted follow-up TVEMs to understand the INT-EXT association for male versus female children (Lanza & Linden-Carmichael, 2021). For our exploratory aim, we repeated the same process to model PCE (0 = no PCE; 1 = any PCE) as a moderator. We centered predictors at each timepoint at the timepoint average value. We completed data preparation and descriptive analyses using IBM SPSS Statistics (Versions 28–29) predictive analytics software. The %TVEM macro (Li et al., 2017) was used to conduct all TVEMs in SAS 9.4 (SAS Institute, Cary, NC) with p-spline estimation (see Supplementary Materials for syntax).

### Attrition and Missing Data

Rates of available data on child behavior problems for the analytic sample across the 8 timepoints varied between 82 and 86%. TVEM uses all available data, with observations excluded using listwise deletion in the presence of missing data on an outcome or predictor/covariate at the assessment level (Lanza & Linden-Carmichael, 2021). Thus, if a child missed one assessment, TVEM would still use all data from this child from other assessments (*n* = 1326 total observations). Using multivariate logistic regression, we found no differences between participants with complete, partially complete, or missing data. See Supplementary Materials for more information.

## Results

### Descriptive Statistics

Table 1 provides descriptive statistics of study variables. Notably, children with PCE exhibited more INT problems at 54 months (*t*[161] = −2.33, *p* =.014) than children in the non-PCE group. Concurrent EXT and INT problems were positively associated at each timepoint (Table 3). Behavioral problems at 18, 24, 30, 36, 42, 48, and 54 months in one domain were positively associated with subsequent problems in the other domain (i.e., at 24, 30, 36, 42, 48, 54, and 60 months). INT and EXT problems were stable over time (Table 3). Descriptive analyses revealed substantially elevated mean scores by school age, particularly for INT and co-occurring problems, with some children having borderline or clinical INT-EXT levels (17.4% for INT problems only and 14.6% for co-occurring and EXT problems compared to < 5% for what we would expect in a random sample; 10.2% for EXT problems only).

**Table 3:** Zero-order correlations among study variables

	2	3	4	5	6	7	8	9	10	11	12	13	14	15	16
**Internalizing Problems**															
1. at 18months	0.67^*^	0.48^*^	0.52^*^	0.41^*^	0.34^*^	0.31^*^	0.44^*^	0.74^*^	0.55^*^	0.48^*^	0.47^*^	0.40^*^	0.35^*^	0.34^*^	0.35^*^
2. at 24 months		0.61^*^	0.69^*^	0.55^*^	0.43^*^	0.47^*^	0.50^*^	0.50^*^	0.73^*^	0.50^*^	0.52^*^	0.46^*^	0.43^*^	0.36^*^	0.43^*^
3. at 30 months			0.67^*^	0.61^*^	0.51^*^	0.51^*^	0.45^*^	0.49^*^	0.51^*^	0.68^*^	0.56^*^	0.55^*^	0.50^*^	0.47^*^	0.46^*^
4. at 36 months				0.73^*^	0.68^*^	0.64^*^	0.52^*^	0.54^*^	0.63^*^	0.60^*^	0.75^*^	0.68^*^	0.66^*^	0.60^*^	0.55^*^
5. at 42 months					0.75^*^	0.63^*^	0.46^*^	0.45^*^	0.51^*^	0.52^*^	0.59^*^	0.73^*^	0.68^*^	0.60^*^	0.54^*^
6. at 48 months						0.63^*^	0.64^*^	0.34^*^	0.41^*^	0.41^*^	0.53^*^	0.61^*^	0.77^*^	0.57^*^	0.61^*^
7. at 54 months							0.54^*^	0.38^*^	0.48^*^	0.44^*^	0.54^*^	0.47^*^	0.59^*^	0.71^*^	0.55^*^
8. at 60 months								0.44^*^	0.40^*^	0.36^*^	0.50^*^	0.43^*^	0.54^*^	0.44^*^	0.70^*^
**Externalizing Problems**															
9. at 18months									0.68^*^	0.65^*^	0.59^*^	0.55^*^	0.49^*^	0.48^*^	0.48^*^
10. at 24 months										0.73^*^	0.69^*^	0.63^*^	0.57^*^	0.59^*^	0.53^*^
11. at 30 months											0.75^*^	0.75^*^	0.65^*^	0.62^*^	0.60^*^
12. at 36 months												0.81^*^	0.78^*^	0.70^*^	0.70^*^
13. at 42 months													0.81^*^	0.72^*^	0.71^*^
14. at 48 months														0.73^*^	0.76^*^
15. at 54 months															0.66^*^
16. at 60 months															

^*^ *p <*.001

### Analyses

**Fig. 1:**
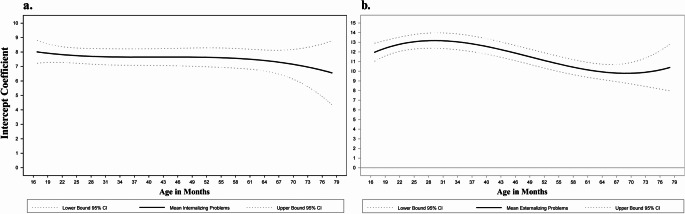
Time-varying mean levels of (a) Internalizing and (b) Externalizing problems. Note: Significance of the mean levels (solidline) of (a) INT and (b) EXT problems over time is determined by examining whether the 95% CI (dotted line) includes 0. INT remained relatively stable, whereas EXT problems followed a curvilinear pattern, with an increase from to 28-30 months, and a stable decrease by 60 months when they stabilized

**Fig. 2:**
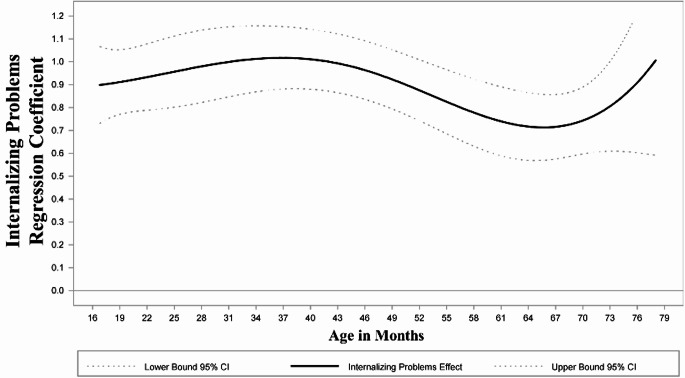
Time-varying effect of internalizing on concurrent externalizing problems. Note: Significance of the time-varying INT-EXT regression coefficient (solid line) is determined by examining whether the 95% CI (dotted line) includes 0 at each age. INT problems were significantly and positively associated with EXT problems in early childhood. INT-EXT association fluctuated over time, increasing from age 16 through 37 months when it was the strongest, and gradually decreasing through 66 months of age when it began increasing again

**Fig. 3:**
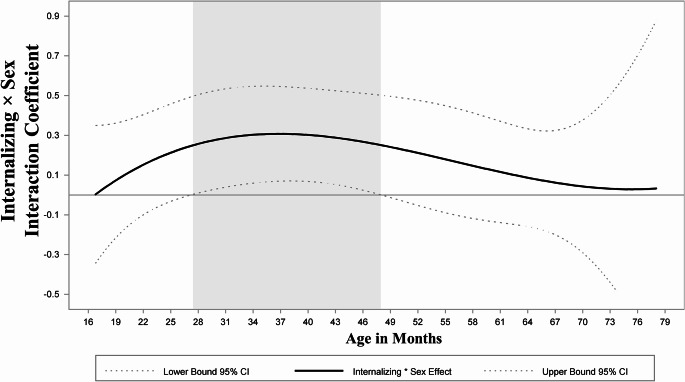
Time-varying internalizing × sex interaction effect on concurrent externalizing problems. Note: Significance of the time-varying interaction effect between INT and sex on EXT (solid line) is determined by examining whether the 95% CI (dotted line) includes zero at each age. The gray area represents the time interval during which 0 was not part of the 95% CIs, indicating a significantINT × sex interaction. Simple slope effects are displayed in Figure 4

**Fig. 4:**
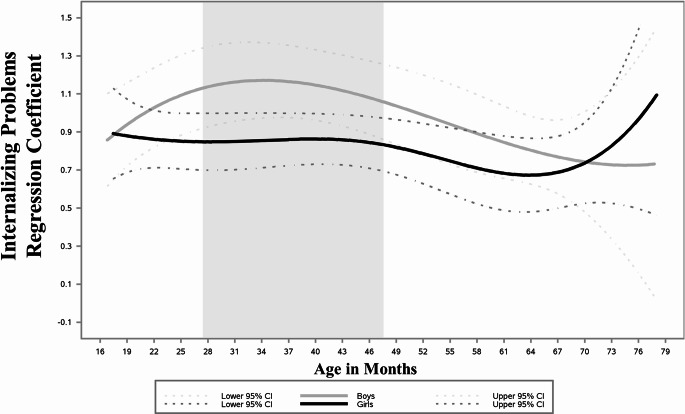
Time-varying interaction effect of internalizing problems for boys (gray) vs girls (black). Note: Male children exhibited a stronger positive INT-EXT effect in late toddlerhood to preschool years (shaded region; age 27–48 months)

**Fig. 5:**
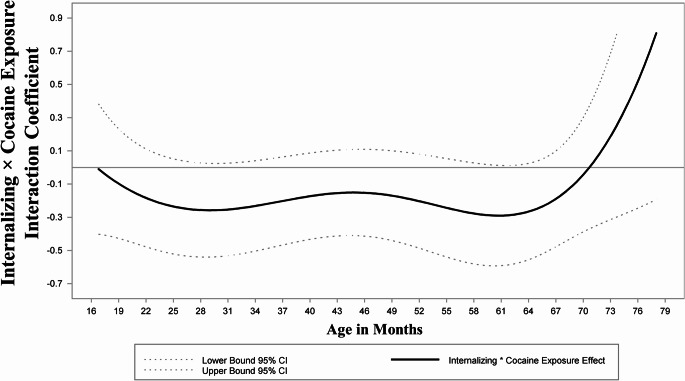
Time-varying internalizing × prenatal cocaine exposure interaction effect on concurrent externalizing problems. Note: Significance of the time-varying interaction effect between INT and prenatal cocaine exposure on EXT (solid line) is determined by examining whether the 95% CI (dotted line) includes zero at each age. There was no significant INT × prenatal cocaine exposure interaction, indicated by 0 being a part of the 95% CIs across ages

First, we examined models that included other prenatal substances and sociodemographic and cumulative environmental risks during the first year of life as covariates (Table 2). As their inclusion did not affect significance, magnitude, or direction of the INT-EXT association, covariates were omitted for parsimony. INT problems remained relatively stable across child age, with a slightly decreasing trend. The average level of EXT problems followed a curvilinear pattern, with an initial increase from 28 months of age, peaking at 30 months, and a decrease by 60 months when EXT problems stabilized. As mean levels of INT and EXT problems followed the same change patterns regardless of whether we controlled for the other problems, we display the mean levels of INT (Fig. 1a) and EXT over time (Fig. 1b), controlling for average levels of the other domain. INT problems were significantly and positively associated with EXT problems in early childhood (Fig. 2). The INT to EXT association fluctuated over time, increasing from age 16 through 37 months when it was the strongest (*b* = 1.02, 95% CI [0.88, 1.16]), and gradually decreasing through 66 months of age (*b* = 0.71, 95% CI [0.57, 0.86]) when it began increasing again. Finally, child’s sex served as a significant moderator of the INT to EXT association (H_2_; Fig. 3). Specifically, compared with female children, males exhibited a stronger positive effect of INT problems on EXT problems in late toddlerhood to preschool years (shaded region; age 27–48 months; Fig. 4). PCE did not moderate the INT-EXT association (Fig. 5).

## Discussion

Our primary goals were to investigate time-varying fluctuations in the INT-EXT association and whether associations differed across early childhood by child sex and PCE. Overall, results indicated that the association of INT with concurrent EXT problems increased from toddler to preschool years, peaked in late toddlerhood, and decreased through early school age, with slight increases at early school age. In addition, we found sex differences, such that compared to female children, male children exhibited a stronger positive effect of INT on EXT problems during the late toddler to preschool years. We did not find any differences in time-varying associations due to the child’s exposure to cocaine during pregnancy.

### Time-Varying Associations between INT and EXT Problems

Consistent with prior literature, on average, children’s EXT problems peaked during the late toddlerhood period (Alink et al., 2006), whereas INT problems remained relatively stable over time (Lavigne et al., 2001). The former finding regarding the peak during late toddlerhood may be particularly important. If INT problems exert a greater influence on EXT problems during the developmental period, this may partially explain the increase in EXT during toddlerhood. Moreover, this finding may support the acting-out explanation for codevelopment (Carlson & Cantwell, 1980; Glaser, 1967). With increased environmental demands and the need for independence, toddlers may experience increased distress (Colson & Dworkin, 1997). As they struggle to express internal distress because of their limited vocabulary and developing cognition (Bjorklund, 2022), they may become more aggressive and defiant. It is possible that the early increase in EXT problems found in the literature may not be due to *pure* EXT problems, but instead indicates dynamic codevelopment between the two problem domains. Thus, targeting INT problems during the earlier developmental stages, when they may be most strongly associated with EXT, may be an avenue for targeting early prevention and intervention efforts for children at risk of developing chronically high co-occurring problems. The current findings underscore the importance of continuing to investigate the nuanced interplay between INT and EXT problems during these developmental stages, since most support for acting-out has been provided by testing sequential directional effects during middle childhood and adolescence (Beyers & Loeber, 2003; Chen & Simons-Morton, 2009).

Nonetheless, it is plausible that the time-varying association between INT and concurrent EXT may not result from an in-the-moment directional effect but from undifferentiated psychopathology and a general predisposition to both. This aligns with the concept of the general *p*-factor (Lahey et al., 2012), suggesting common etiological influences that may not be fully captured. However, based on prior literature, we would anticipate the association to either increase or remain stable over time (Murray et al., 2016). Our results did not follow this pattern during early childhood but instead displayed a predominantly curvilinear pattern. It is possible that sample characteristics or specific developmental ages influenced our results, as previous studies have primarily examined these changes at later ages (Murray et al., 2016). For example, compared with lower-risk samples (Bongers et al., 2003; Gilliom & Shaw, 2004), a larger proportion of behavior problem mean scores were elevated by school age, particularly for INT and co-occurring problems, with some children having borderline or clinical INT-EXT levels. While these are descriptive data and not statistical comparisons, they contextualize the results, indicating generally higher rates of INT and co-occurring problems for children in the current sample at early school age, a time when there are normative declines in EXT and generally low-to-moderate levels of INT (Alink et al., 2006; Lavigne et al., 2001). Last, as TVEM provides a regression coefficient rather than shared variance scores, future research could also explore how actual p-factor scores rather than INT-EXT associations vary over time.

### Sex Differences in Time-Varying Associations

We found that the association between INT and EXT problems was stronger for males than females in late toddlerhood to preschool years (age 27–48 months). While gender socialization processes are likely to have affected this association, it is challenging to disentangle biological and acculturation/gender socialization effects, as we did not directly measure these constructs (Brody, 2000; Chaplin & Aldao, 2013; Endendijk et al., 2018). Gender socialization theories suggest that children are socialized to express their distress and emotions in a gender-congruent manner based on their sex at birth, and gender-incongruent behaviors may result in more negative environmental responses (Endendijk et al., 2018; Ostrov & Godleski, 2010). In addition, Loeber and Keenan (1994) proposed that when displaying problems in a domain with less prevalence for one of the sexes (e.g., male children displaying INT), the effect on the other domain is stronger than when displaying problems in the domain with higher prevalence (e.g., male children displaying EXT). Our results may partially support this hypothesis, with a significant association between INT and concurrent EXT problems among male children (Moffitt et al., 2001). However, similar to most research on the ‘gender paradox,’ the abovementioned studies have been conducted in middle childhood and adolescence (e.g., Wiesner & Kim, 2006). Our findings address the particular gap in these codeveloping differences in early childhood, especially in a sample at elevated risk. Future research could investigate specific mechanisms by assessing gender socialization measures to better explain the sex differences we found.

Another potential explanation beyond the scope of the current study is the role of prenatal influences differing by child sex. Specifically, researchers have commonly observed sex differences in the effects of prenatal exposure. For example, PCE has been associated with elevated EXT problems in boys compared to girls (e.g., Delaney-Black et al., 2004), whereas in others, PCE has been associated with elevated behavioral problems in girls only (Finger et al., 2022; Minnes et al., 2010). It is possible that the associations between INT and EXT problems may differ by PCE depending on child’s sex. However, our findings did not change when PCE was included in the model examining differences by sex. As we did not have a sufficient sample size to examine a three-way interaction between PCE, sex, and child age, future studies with larger sample sizes are required to investigate these complex associations.

### Prenatal Cocaine Exposure Differences in Time-Varying Associations

To our knowledge, this is the first study to explore time-varying associations between INT and EXT problems as a function of PCE. We did not find that PCE moderated the association between INT and EXT. As this was a mostly exploratory aim, we did not have a-priori hypothesis for this relationship. However, there is rich literature on the effects of PCE on EXT problems (Bendersky et al., 2006; Minnes et al., 2010; Richardson et al., 2011) and emotional, cognitive, and biological dysregulation (Ackerman et al., 2010; Eiden et al., 2014; Schuetze et al., 2020). Thus, it was possible to expect that during the early stages, when children are still developing cognition and self-regulation, they would be more likely to express their internal distress as EXT (per the acting-out explanation). However, we did not find differences in INT-EXT associations by PCE group.

Another explanation is that past evidence showing links between PCE and EXT is because PCE is only a predictor of pure EXT problems rather than their co-occurrence with INT. In this scenario, we may expect to find cross-lagged relationships per the failure explanation by accumulating experiences of academic and social difficulties. To examine such relationships, we considered time-lagging INT and EXT problems. However, there are two major methodological considerations. First, although we attempted to assess all families at 6-month intervals, there were variations around the exact child age at each assessment point. Therefore, even if the child completes all assessments, the gap between timepoints will vary somewhat when using precise age, making the interpretation of the concurrent association difficult. Second, even if we proceed with lagging time, we will then face additional issues with a high percentage of missing data due to the listwise deletion procedure at the assessment level. In particular, with the lag of one timepoint, each child’s *Y* outcome will now depend on the availability of *X* at the prior timepoint (e.g., child’s EXT problems at 24 months will only be included in the model if the child had report of INT at 18 months). Based on those methodological limitations, we decided to retain the continuous age. Future research could investigate cross-lagged associations as a function of PCE and other substances, using more traditional models, such as random-intercept cross-lagged panel model.

Lastly, in addition to the fetal stress posed by exposure to substances such as cocaine, PCE may serve as a marker of increased environmental risks, such as harsher parenting, violence exposure, continued exposure to substances, low caregiver psychological functioning, and instability in the home environment (Eiden et al., 2014; Seay et al., 2023), all predictive of higher levels of behavior problems (e.g., Perry et al., 2021). While we tested models that included many of these covariates, they did not change the significance, magnitude, or direction of the INT-EXT association and were omitted for parsimony. Future studies could examine them as moderators of prenatal risk using larger samples and alternative analytical approaches.

### Strengths and Limitations

We acknowledge that our study had several limitations. First, the assessment of INT-EXT problems was based on caregiver reports only. The depression-distortion hypothesis provides evidence that caregivers who experience high levels of depression and other psychopathologies may provide biased reports of child behavior (Richters & Pellegrini, 1989). While we found identical patterns in the time-varying effects when including composite measures that included caregivers psychological functioning as a covariate, future research may benefit from utilizing reports from other caregivers and teachers. However, it is important to recognize that many of the caregivers in the current sample were single parents, and not all children may attend childcare prior to entering the public school system; thus, this may be more difficult in high-risk samples. Second, the sample primarily included Black families with lower socioeconomic status who experienced higher levels of prenatal and contextual risks; thus, the generalizability of the results is limited to this population. Future research could examine whether these relationships differ across children from diverse families who are not biologically vulnerable to prenatal substance exposure. Third, although we were able to test the effect of PCE, we used a dichotomous indicator of any versus no PCE. We made this choice to capitalize on both biological and self-report measures, as well as our conceptualization of the role of PCE as a marker for other co-occurring environmental risks and polysubstance use. However, prior literature has indicated mixed findings on the effect of PCE on early behavioral problems when using categorical versus continuous measures of PCE (Eiden et al., 2014; Lin et al., 2018). Thus, future studies could investigate whether a dose-response effect exists across development.

Fourth, some researchers argue that behavior problems may be undifferentiated during early childhood (Oland & Shaw, 2005; Willner et al., 2016), making it challenging to measure INT and EXT problems separately. While the undifferentiated distress mechanism has not been extensively tested, newer analytical approaches, such as latent transition analysis, offer opportunities to examine whether groups of young children with co-occurring problems transition into more distinct phenotypes. However, while some children may shift from co-occurring to more pure problem expressions (i.e., differentiation), studies have also found that co-occurring and pure profiles tend to remain relatively stable over time (e.g., Isdahl-Troye et al., 2022), supporting the idea that these are distinct domains. Furthermore, the CBCL’s internalizing and externalizing domains have demonstrated high reliability and validity in children, including those aged 0–5 years (Halle & Darling-Churchill, 2016). Related to this issue, some research has found some bias in internalizing and externalizing scales based on child age (Zheng et al., 2024), while others have demonstrated longitudinal measurement invariance in early childhood (Colder et al., 2002). While the issue of longitudinal measurement invariance is usually utilized in latent variable models and TVEM does not allow for such invariance testing, more testing on these measures in early childhood should be done in future work to ensure that our time-varying effects are not partially due to measurement over time.

Finally, while examining INT and EXT problems allows us to investigate nuances in these complex dynamic relationships compared to examining them separately or as a total score, the two broadband constructs remain heterogeneous. Future studies could examine these dynamic relationships using specific subscales of INT (e.g., emotionally reactive, anxious/depressed, withdrawn) and EXT problems (e.g., attention and aggressive problems; Achenbach & Rescorla, 2000). Future studies could also incorporate individual child characteristics that may more strongly affect one or both domains during sensitive age periods.

Despite these limitations, our paper has several strengths, such as its longitudinal prospective design with frequent INT-EXT measurements during early childhood, a sample consisting of underrepresented families experiencing high levels of pre- and postnatal adversities, and high retention rates over time. The use of a multi-method assessment of PCE, including well-validated and intensive calendar-based self-report methods, biomarkers, and medical record reviews, was an additional strength. Additionally, our analytical approach allowed us to examine not only differences in the nature of the association between INT and EXT problems across time, but also how this relationship may vary by child sex and PCE. This method addresses some limitations posed by traditional approaches (Burt & Roisman, 2010; Fanti & Henrich, 2010; Gilliom & Shaw, 2004) by allowing non-parametric estimation of effects and detection of nuanced changes in the magnitude and direction of associations.

### Conclusion

Identifying sensitive periods when the relationships between INT and EXT problems are the most pronounced allows for targeted interventions. With growing evidence of the dynamic interplay between INT and EXT problems, we contribute to the literature by utilizing TVEM to examine the nuances of changes in the magnitude and direction of INT-EXT associations. Our findings are in line with the acting-out explanation of codevelopment during early childhood and may serve as a potential explanation for the observed increases in EXT problems during this developmental stage, especially in male children. While we did not find any significant associations by PCE status, this was the first study to examine fluctuating codeveloping INT and EXT associations as a function of PCE. Future work should continue to investigate etiologic factors associated with individual differences in time-varying INT-EXT associations and important protective factors so that they can be individually targeted in prevention programs, during critical timing of codeveloping dynamics where one behavior problem (INT) may have more impact on the other (EXT).

## Supplementary Information

Below is the link to the electronic supplementary material.

Supplementary Material 1 (DOCX 24.3 KB)

Supplementary Material 2 (DOCX 20.3 KB)

## Data Availability

Data supporting this study are available from the second author upon reasonable request. Access to the data is subject to approval and a data sharing agreement due to the sensitive nature of substance use during pregnancy.
