# Supplementary material for: From Distress To Disruption in Early Childhood: Time-Varying Associations Between Internalizing and Externalizing Problems, Child Sex and Prenatal Cocaine Exposure
Source: Res Child Adolesc Psychopathol. 2025 Oct 21;53(12):1971–86. doi: 10.1007/s10802-025-01369-z (PMC12718271; doi:10.1007/s10802-025-01369-z)
Supplement: Supplementary file 1 — Supplementary Material 1 (DOCX 24.3 KB) [file 10802_2025_1369_MOESM1_ESM.docx]

**Method**

**Measures**

***Potential Covariates***

The following variables were used as covariates based on previous literature including previous results from the current sample (e.g., Authors, 2007, 2011, 2020). We planned to test models where we controlled for other prenatal substance exposure, cumulative environmental and sociodemographic risks during the first year of child's life.

**Other Prenatal Substance Exposure**. Prenatal substance exposure was determined by aggregating use of alcohol, marijuana, and tobacco during pregnancy from self-report. Similar to prenatal cocaine exposure, other prenatal substance exposure was measured using the TLFB (Sobell et al., 1986), yielding the average number of joints smoked per week, the average number of cigarettes smoked per week, and the average number of standard drinks per week during the entire pregnancy. Proportion scores were calculated by taking the average weekly amount for cannabis, cigarettes, and alcohol used per the self-report and dividing it by the total number of days or maximum amount used during pregnancy for each substance. Higher values represent higher levels of other than cocaine prenatal substance exposure.

**Environmental Risk.** In a manner similar to Authors (2020), cumulative environmental risk score was computed from measures assessing caregiver psychological functioning (Brief Symptom Inventory [BSI]; Derogatis, 1993), caregiver exposure to violence (TLFB; Fals-Stewart et al., 2003), caregiving instability (Structured Clinical Interview [SCI]; Platzman et al., 2001), and caregiver postnatal substance use (TLFB; Sobell et al., 1986). Risk was first individually dummy coded for each variable at each of the 1-, 7-, and 13-months assessments with code of 1 reflecting risk in each domain: 1) if the caregiver had lower psychological functioning (BSI score was one SD above the sample mean); 2) if the caregiver was exposed to any violence; 3) if there were two or more experiences of caregiver instability (e.g., separations from primary caregiver, changes in caregiving adults and living environment); 4) if the caregiver had a substance use score greater than 10 cigarettes per day for tobacco, 4 drinks per day for alcohol, 1 joint per day for cannabis, and any cocaine (separate score for each substance) reflecting continued high risk postnatal substance exposure. The final cumulative environmental risk score was a sum of the individual 30 dummy-coded risk scores. Higher values represent higher cumulative environmental risk.

**Sociodemographic Risk.** We calculated a cumulative sociodemographic risk score (caregiver race, low caregiver education, single parenthood, low income-to-needs ratio, and occupational status) to account for the multiple risks. Following Author (2019) approach, we calculated a cumulative risk score, operationalizing sociodemographic risk as aggregation across risk factors as these risks factors commonly co-occur (Evans et al., 2013; Wilson et al., 2009). In addition, caregiver non-white race or ethnicity was included in the cumulative sociodemographic risk score to serve as a proxy for structural barriers, disparities, racial discrimination, and bias faced by people of color. Information about caregiver race, caregiver education, caregiver partner status, caregiver income-to-needs ratio, and caregiver occupational status were collected during the first lab visit (4-8 weeks of infant age). Caregiver occupational status was coded using the Hollingshead scale (*M* = 3.06, *SD* = 1.60, range = 1–9; Hollingshead, 1975). Risk was first individually dummy coded for each variable with a code of 1 reflecting risk: 1) if caregiver was non-white (84.2%); 2) if caregiver had not received a high school diploma or equivalent (39.5%); 3) if caregiver was never married (66%); 4) if caregiver had an occupation status in lower quartile (41.2%); 5) if caregiver had an income-to-needs ratio in lower quartile (25.4%). The final sociodemographic cumulative risk variable was a sum of the individual dummy-coded risk scores. Higher values represent higher sociodemographic risk.

**Analytic Plan**

TVEM was used to address our study aims (Lanza & Linden-Carmichael, 2021). TVEM is an extension of linear regression that examines the dynamic nature of associations over time. TVEM allows the intercept and regression coefficients to vary flexibly as nonparametric functions of time. For example, to examine the time-varying effect of internalizing problems on concurrent externalizing problems across child age, the model was estimated as:

*EXTi = β0 (t) + β1(t)INTi + ∈i ,*

Here, the intercept (*β_0_*) represents the mean level of externalizing problems across time, accounting for average internalizing problems, and *β_1_* represents the regression coefficient function for the time-varying effects of internalizing problems on externalizing problems during early childhood, controlling for prenatal cocaine exposure. Finally, to examine the moderating effect of child's sex, we performed time-varying moderation, estimated as:

*EXT_i_* = *β_0_* (*t*) + *β_1_*(*t*)*INT_i_* + *β_2_*(*t*)*SEX_i_* + *β_3_*(*t*)*INT*SEX_i_* + *∈_i_* ,

where we respectively examined whether the effect of internalizing problems on externalizing problems varied as a function of child's sex over time (0 = female; 1 = male). As outlined in Lanza and Linden-Carmichael (2021), similar to standard practices in linear regression, if an interaction term was significant at any timepoint, we would conduct follow-up models to test the effect of sex on the association between internalizing and externalizing problems. Significant effects were determined by examining whether the 95% confidence intervals included zero at any point across time (Lanza & Linden-Carmichael, 2021). To address our exploratory aim, we repeated the same process to model prenatal cocaine exposure (0 = no prenatal cocaine exposure; 1 = any prenatal cocaine exposure) as the moderator.

Predictors were centered for each timepoint at the timepoint average. Data preparation and descriptive analyses were completed in IBM SPSS Statistics (Versions 28-29) predictive analytics software. All TVEMs were conducted in SAS 9.4 (SAS Institute, Cary, NC) using the *%TVEM* macro (Li et al., 2017). P-spline estimation was used in all TVEM analyses. The syntax for the main effect and interaction models are provided below.

**Attrition and Missing Data**

Out of the children included in final analyses (*n =* 198), the following were rates for complete data on child behavior problems: 84% (18 months), 86% (24 months), 85% (30 months), 83% (36 months), 83% (42 months), 82% (48 months), 82% (54 months), and 84% (early school age). It is important to note that TVEM uses all available data, with observations excluded using listwise deletion if there is missing data on an outcome or predictor/covariate at the level of the assessment (Lanza & Linden-Carmichael, 2021). For example, if a child missed one assessment, TVEM will utilize all available data from this child from other assessments. Given that estimation biases can arise if participants who contribute data to the model at every point in time are not representative of the full sample (Shiyko et al., 2014), we conducted attrition analyses to determine whether participants who were assessed at all occasions differed from those with missing data on any occasion in terms of externalizing and internalizing problems, prenatal cocaine exposure, and child sex. Results from multivariate logistic regression analysis indicated that there were no significant differences between participants with complete versus missing data. Thus, we used all available data from all participants regardless of missingness. As TVEM estimates associations between variables as a continuous function of time, we also examined whether there is adequate coverage of data for externalizing and internalizing problems across the entire time axis (sample range: 16–78 months of child age) when combining across both people and assessments. Examination of sample sizes showed that there are 1326 total assessments, indicating there is adequate data on children's externalizing and internalizing problems across time.

**Results**

**Descriptive Statistics**

Table 1 in the main manuscript provides descriptive statistics of study variables and differences in study variables by child sex and prenatal cocaine exposure with behavior problems at each timepoint. Notably, children in the cocaine-exposed group exhibited more internalizing problems at 54 months (*t*[161] = -2.33, *p* = .014) than children in the control group.

Table 3 in the main manuscript displays zero-order correlations between externalizing and internalizing problems and the stability of these variables over time. Concurrent externalizing and internalizing problems were positively associated from 18 to 60 months (see Table 2 dark gray boxes). Externalizing problems at 18, 24, 30, 36, 42, 48, and 54 months were positively associated with subsequent internalizing problems (i.e., at 24, 30, 36, 42, 48, 54, and 60 months) and internalizing problems at 18, 24, 30, 36, 42, 48, and 54 months were positively associated with subsequent externalizing problems (i.e., at 24, 30, 36, 42, 48, 54, and 60 months). Internalizing and externalizing problems showed high stability across time (see Table 2 white boxes).

**Preliminary Analyses**

All initial TVEMs included the following covariates specifying time-invariant and time-variant effects: prenatal cocaine exposure, other prenatal substance exposure (marijuana, tobacco, alcohol), socio-demographic factors (race and ethnicity, lower income and education, single parent status, lower occupation status), and cumulative environmental risks during the first year of child's life (continued substance use, exposure to violence, lower caregiver psychological functioning, caregiving instability). These covariates did not change the significance, magnitude, or direction of associations across all models. Thus, the covariates were omitted for parsimony.
