# Supplementary material for: From Distress To Disruption in Early Childhood: Time-Varying Associations Between Internalizing and Externalizing Problems, Child Sex and Prenatal Cocaine Exposure
Source: Res Child Adolesc Psychopathol. 2025 Oct 21;53(12):1971–86. doi: 10.1007/s10802-025-01369-z (PMC12718271; doi:10.1007/s10802-025-01369-z)
Supplement: Supplementary file 2 — Supplementary Material 2 (DOCX 20.3 KB) [file 10802_2025_1369_MOESM2_ESM.docx]

**Variable names:**

EP = Externalizing problems (non-centered at the timepoint assessment)

IP = Internalizing problems (non-centered at the timepoint assessment)

EPc = Externalizing problems (centered at the timepoint assessment)

IPc = Internalizing problems (centered at the timepoint assessment)

AGEm = precise age in months calculated from birthdate to assessment date

x0 = intercept (= 1 for everyone)

SEX = biological sex (female sex = 0, male sex = 1)

SexXepC = sex x centered externalizing problems interaction term

SexXipC = sex x centered internalizing problems interaction term

PCE = prenatal cocaine exposure (no exposure = 0, exposure = 1)

CocXepC = cocaine exposure x centered externalizing problems interaction term

CocXipC = cocaine exposure x centered internalizing problems interaction term

**Model notations:**

dist = distribution

time = time variable

dv = dependant variable

tvary_effect = time-varying effect variable (intercept and independent variables)

knots = a measure of flexibility, the number to be used in estimating each time-varying coefficient

*******************************************************

* MODEL 1: EP -> IP *

******************************************************;

* FULL MODEL: time-varying effect of EP on IP;

%***TVEM***(Method = p-spline,

dist = normal,

data = subset,

id = ID,

time = AGEm,

dv = IP,

tvary_effect = x0 EPc,

random = none,

knots = **10** **10**);

* FULL MODEL: time-varying effect of EP on IP, moderation by Sex;

%***TVEM***(data = subset,

time = AGEm,

id = ID,

dv = IP,

tvary_effect = x0 EPc SexXepC SEX,

knots = **10** **10** **10** **10**,

method = p-spline,

dist = normal);

* FULL MODEL: time-varying effect of EP on IP, moderation by prenatal cocaine exposure;

%***TVEM***(data = subset,

time = AGEm,

id = ID,

dv = IP,

tvary_effect = x0 EPc PCE CocXepC,

knots = **10** **10** **10** **10**,

method = p-spline,

dist = normal);

*******************************************************

* MODEL 2: IP -> EP *

******************************************************;

* FULL MODEL: time-varying effect of IP on EP;

%***TVEM***(data = subset,

time = AGEm,

id = ID,

dv = EP,

tvary_effect = x0 IPc,

knots = **10** **10**,

method = p-spline,

dist = normal);

* FULL MODEL: time-varying effect of IP on EP, moderation by Sex;

%***TVEM***(data = subset,

time = AGEm,

id = ID,

dv = EP,

tvary_effect = x0 IPc SEX SexXipC,

knots = **10** **10** **10** **10**,

method = p-spline,

dist = normal);

* FULL MODEL: time-varying effect of IP on EP, moderation by prenatal cocaine exposure;

%***TVEM***(data = subset,

time = AGEm,

id = ID,

dv = EP,

tvary_effect = x0 IPc PCE CocXipC,

knots = **10** **10** **10** **10**,

method = p-spline,

dist = normal);
